# RNA-Seq transcriptome data of human cells infected with influenza A/Puerto Rico/8/1934 (H1N1) virus

**DOI:** 10.1016/j.dib.2020.106604

**Published:** 2020-11-29

**Authors:** Evgenii Zhuravlev, Mariia Sergeeva, Sergey Malanin, Rinat Amirkhanov, Dmitriy Semenov, Tatiana Grigoryeva, Andrey Komissarov, Grigory Stepanov

**Affiliations:** a Institute of Chemical Biology and Fundamental Medicine, Siberian Branch of the Russian Academy of Sciences, Novosibirsk, Russia; b Smorodintsev Research Institute of Influenza, Ministry of Health of the Russian Federation, St. Petersburg, Russia; c Institute of Fundamental Medicine and Biology, Kazan Federal University, Kazan, Russia

**Keywords:** RNA-seq, Influenza A virus, H1N1, Human cells, Transcriptome

## Abstract

Human influenza remains a serious public health problem. This data article reports the transcriptome analysis data of human cell lines infected with influenza A/Puerto Rico/8/1934 (H1N1) virus. Mock-infected cells were included as controls. Human embryonic fibroblasts (MRC-5) and immortalized cell lines (A549, HEK293FT, WI-38 VA-13) were selected for RNA sequencing using Illumina NextSeq500 platform. Raw data were applied to the bioinformatic pipeline, which includes quality control with FastQC and MultiQC, adapter and quality trimming with Cutadapt, filtering to the genome of influenza A with STAR, transcript quantification with Salmon tool (GRCh38_RefSeq_Transcripts). Differential expressed genes were identified using R package DESeq2 with FDR-adjusted p-value < 0.001 and absolute value of log2(FC) > 1. Lists of differentially expressed genes is provided. The raw and processed RNA-seq data presented in this article were deposited to the European Nucleotide Archive via the ArrayExpress partner repository with the dataset accession number E-MTAB-9511 .

## Specifications Table

**Table:** 

Subject	Molecular biology, Biochemistry, Virology
Specific subject area	NGS, Transcriptomics
Type of data	FASTQ files Processed data files Table Chart
How data were acquired	Sequencing data acquired by Illumina NextSeq 500 platform (1 × 75 bp single-read sequencing)
Data format	Raw and Processed Data
Parameters for data collection	MRC-5, WI-38 VA-13, A549 and HEK293FT human cells were infected with influenza A/Puerto Rico/8/1934 virus at a dose of 1 TCID50/cell and incubated at 37°С for 48 h. Mock-infected cells were included as controls.
Description of data collection	Following the incubation period, the cell monolayer was directly lysed. Total RNA was extracted, then polyA RNA fraction was enriched and used for construction cDNA libraries using a NEBNext Ultra II Directional RNA library preparation kit and NEBNext mRNA Magnetic Isolation Module. Massive parallel sequencing was performed on a NextSeq Illumina 500 platform using NextSeq 500/550 High Output v2.5 Kit (75 cycles).
Data source location	Institute of Chemical Biology and Fundamental Medicine, Siberian Branch of the Russian Academy of Sciences, Novosibirsk, Russia
Data accessibility	Both raw and processed RNA-seq data were deposited to the European Nucleotide Archive via the ArrayExpress partner repository with the dataset accession number E-MTAB-9511. Repository name: ArrayExpress Data identification number: E-MTAB-9511 Direct URL to data: https://www.ebi.ac.uk/arrayexpress/experiments/E-MTAB-9511/

## Value of the Data

• The analysis of differentially expressed genes across MRC-5, WI-38, A549 and HEK293FT cell lines infected with influenza A virus may contribute to a better understanding mechanisms of cell permissiveness to influenza infection.
• Raw FASTQ files available in the ArrayExpress repository can be processed by researchers using their own bioinformatic pipelines or analyzed as part of a large combined data sets for extended statistical analysis.
• The comparison of the several cell lines may allow for the selection of cell lines for isolation and propagation of seasonal strains and production of vaccine strains.
• The identification of activated or deactivated molecular metabolic and signaling pathways in infected cells may indicate the direction for future research in the field of antiviral therapy.

## 1 Data Description

**Fig. 1:**
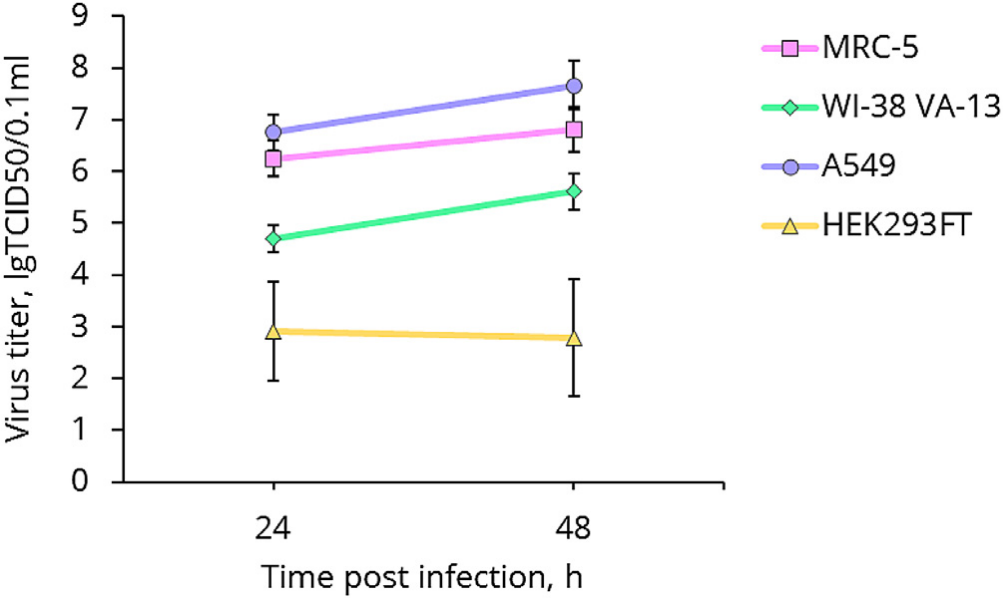
Growth kinetics of influenza A/Puerto Rico/8/1934 (H1N1) virus in MRC-5, WI-38 VA-13, A549 and HEK293FT cell lines. At the indicated time points post infection, virus titres were determined by use of plaque assays in MDCK cells. Values shown are the means (±s.d.) of three independent experiments.

**Fig. 2:**
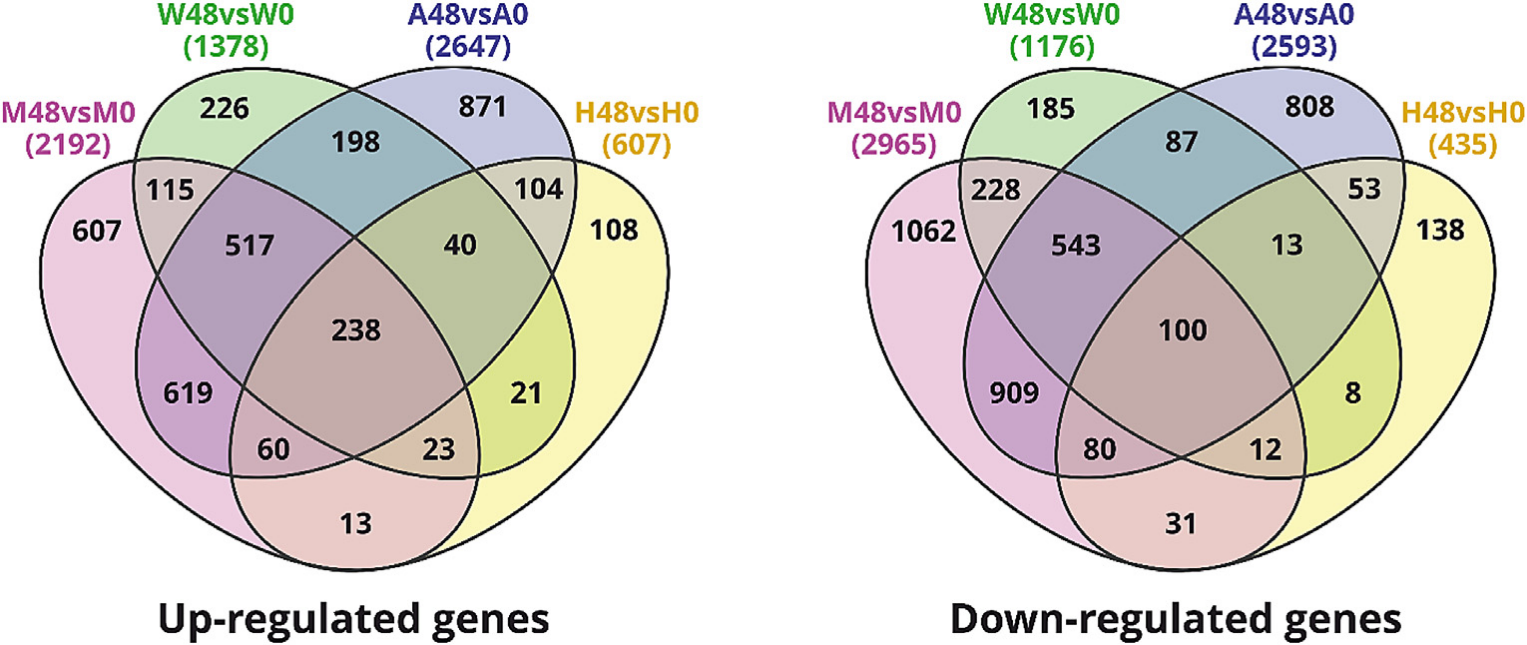
Comparison of differentially expressed genes. Venn diagrams show the number of common and unique up-regulated and down-regulated DEGs of MRC-5 (M48vsM0), WI-38 VA-13 (W48vsW0), A549 (A48vsA0) and HEK293FT (H48vsH0) cell lines, 48 h after influenza infection.

To study genes involved in cellular response to Influenza A virus infection, the transcriptome analysis of MRC-5, WI-38 VA-13, A549 and HEK293FT human cell lines infected with influenza A/Puerto Rico/8/1934 (H1N1) virus was performed on the Illumina NextSeq500 platform. The raw data (FASTQ files) were deposited to the European Nucleotide Archive via the ArrayExpress partner repository with the dataset accession number E-MTAB-9511 [1]. Growth kinetics of influenza A/Puerto Rico/8/1934 (H1N1) virus in cell lines are presented in Fig. 1. Names of cell lines samples, statistics of raw, trimmed and filtered reads are shown in Table 1. The tab-delimited text files (*.txt) which have been produced after transcript quantification with Salmon tool (GRCh38_RefSeq_Transcripts) were also uploaded on repository. Lists of differentially expressed genes were obtained using R package DESeq2 with FDR-adjusted *p*-value < 0.001 and absolute value of log2(FC) > 1 (Supplementary Table 1). Venn diagrams showing comparison of lists of up-regulated and down-regulated DEGs between MRC-5, WI-38 VA-13, A549 and HEK293FT cell lines are presented in Fig. 2.

**Table 1:** Number of raw, trimmed and filtered reads of 16 cDNA libraries of mock-infected (0h) and infected with influenza A virus (48h) cell lines (MRC-5, WI-38 VA-13, A549 and HEK293FT).

Sample name	Biological replicate	M Reads (Raw)	M Reads (after trimming)	M Seqs (after filtering)
MRC-5_0h	M01	11.3	11.3	11.3
	M02	11.6	11.6	11.6
MRC-5_48h	M481	11.4	11.3	10.0
	M482	11.5	11.4	10.1
WI-38 VA-13_0h	W01	13.8	13.8	13.8
	W02	13.9	13.9	13.9
WI-38 VA-13_48h	W481	13.9	13.9	12.6
	W482	14.1	14.1	12.4
A549_0h	A01	12.7	12.7	12.7
	A02	10.6	10.6	10.6
A549_48h	A481	11.4	11.4	8.9
	A482	11.6	11.5	8.9
HEK293FT_0h	H01	13.0	13.0	13.0
	H02	13.6	13.6	13.6
HEK293FT_48h	H481	14.1	14.1	13.7
	H482	13.5	13.4	12.7

## 2 Experimental Design, Materials and Methods

### 2.1 Virus and cell lines

A/Puerto Rico/8/1934 (H1N1) influenza virus from the collection of Smorodintsev Research Institute of Influenza (Ministry of Health of the Russian Federation) was used in the experiments.

MRC-5 (ATCC #CCL-171, derived from ATCC, USA) and WI-38 VA-13 (ATCC #CCL-75.1, derived from Russian Collection of Cell Cultures, Russia) cells were maintained in MEM (Gibco, USA) supplemented with GlutaMAX (Gibco, USA), sodium pyruvate (Gibco, USA) and 10% fetal bovine serum (FBS) (Gibco, USA) at 37 °C, 5% CO_2_. A549 cells (ATCC #CCL-185, derived from ATCC, USA) were maintained in DMEM/F12 (Gibco) supplemented with GlutaMAX and 10% FBS at 37 °C, 5% CO_2_. HEK293FT cells (Cat# R70007, Thermo Fisher Scientific, USA) were maintained in DMEM/F12 supplemented with GlutaMAX, 10% FBS and 100 U/mL Penicillin-Streptomycin (Gibco, USA) at 37 °C with 5% CO_2_.

### 2.2 Infection of cells and growth kinetics of influenza virus

Cells were grown in T25 cell culture flasks (TPP, Switzerland) until 90–100% monolayer, then infected with influenza A/Puerto Rico/8/1934 virus at a dose of 1 TCID50/cell (two replicates), and incubated at 37 °С for 48 h. At 24 and 48 h after infection, all the culture media were collected, and the titers were measured by plaque assay. Mock-infected cells were included as controls. Following the incubation period, the cells were washed twice with PBS and directly lysed by adding LIRA reagent (Biolabmix, Russia).

### 2.3 RNA isolation

Total RNA was extracted from cells with LRU RNA extraction kit (Biolabmix, Russia) following the manufacturer's protocol. RNA concentration was assessed using the Qubit 2 fluorometer (Thermo Fisher Scientific, USA) with Qubit RNA HS Assay Kit (Thermo Fisher Scientific, USA). The quality of total RNA expressed as RNA Integrity Number (RIN) was determined with Bioanalyzer 2100 instrument (Agilent, USA) using an Agilent RNA Pico 6000 Kit (Agilent, USA) [2]. The threshold RIN reading greater than 7.0 was taken as cut-off point for transition to the stage of library preparation.

### 2.4 Library preparation and sequencing

A total of 16 cDNA libraries were prepared from two biological replicates of each time point (0 h (mock-infected) and 48 h MRC-5, WI-38 VA-13, A549 and HEK293FT). The construction of cDNA libraries according to a standard protocol using a NEBNext Ultra II Directional RNA library preparation kit (New England Biolabs, UK) and NEBNext mRNA Magnetic Isolation Module (New England Biolabs, UK), as well as massive parallel sequencing on a NextSeq Illumina 500 platform, were conducted at the Institute of Fundamental Medicine and Biology, Kazan Federal University (Kazan, Russia). For the isolation of mRNA, fragmentation and priming procedure 1 µg of the total RNA was used. A NextSeq 500/550 High Output v2.5 Kit (75-nucleotide single-end reads) (Illumina, US) was used. For the prepared sequencing libraries, fragment size distribution was analysed using Bioanalyzer 2100 instrument (Agilent, USA) using an Agilent High Sensitivity DNA Kit (Agilent, USA) and quantification by the Qubit 2 fluorometer (Invitrogen, USA) with Qubit DNA HS Assay Kit (Thermo Fisher Scientific, USA). Fragment size range between 250 bp to 700 bp with clear peak on 300 bp was observed.

### 2.5 RNA-seq analysis

The raw data were saved as FASTQ format files. The quality control of the raw and trimmed reads was performed using FastQC and MultiQC [3,4]. Trimming of the adapter content and Quality trimming was performed using Cutadapt [5]. The reads complementary to the genome of influenza A/Puerto Rico/8/1934 (H1N1) were filtered out from the trimmed reads using STAR [6]. The filtered reads were used for transcript quantification by Salmon tool (GRCh38_RefSeq_Transcripts) [7]. The R-package Tximport was used to convert the transcript quantifications to gene quantifications [8].

### 2.6 Differential expression analysis

To study genes involved in cellular response to influenza A virus infection, differential expressed genes were identified using R package DESeq2 with a FDR-adjusted *p*-value < 0.001 and the absolute value of a log2(FC) > 1 [9]. Overall, 2192, 1378, 2647 and 607 genes were up-regulated and 2965, 1176, 2593 and 435 genes were significantly down-regulated in MRC-5, WI-38 VA-13, A549 and HEK293FT cells, respectively. Of these, 238 common genes were up-regulated while 100 common genes were down-regulated in all cell lines.

## Credit Author Statement

**Evgenii Zhuravlev:** Software, Formal analysis, Data Curation, Visualization, Writing - Original Draft; **Mariia Sergeeva:** Methodology, Investigation, Writing - Review & Editing; **Sergey Malanin:** Investigation; **Rinat Amirkhanov:** Methodology; **Dmitriy Semenov:** Software, Supervision; **Tatiana Grigoryeva:** Supervision; **Andrey Komissarov:** Conceptualization; **Grigory Stepanov:** Conceptualization, Methodology, Project administration and Funding acquisition.

## Declaration of Competing Interest

The authors declare that they have no known competing financial interests or personal relationships which have, or could be perceived to have, influenced the work reported in this article.

## Appendix Supplementary materials

Image, application 1
